# Production of a recombinant capsid protein VP1 from a newly described polyomavirus (RacPyV) for downstream use in virus characterization

**DOI:** 10.1016/j.dib.2016.01.070

**Published:** 2016-02-09

**Authors:** Molly E. Church, Florante N. Dela Cruz, Kevin Kim, Michele Persiani, Leslie W. Woods, Patricia A. Pesavento, Kevin D. Woolard

**Affiliations:** a UC Davis, School of Veterinary Medicine, Department of Pathology, Microbiology, and Immunology, USA; b UC Davis, School of Medicine, Department of Dermatology, USA; c UC Davis, School of Veterinary Medicine, California Animal Health and Food Safety Laboratory System, USA

## Abstract

Here we describe the methods for production of a recombinant viral capsid protein and subsequent use in an indirect enzyme linked immunosorbent assay (ELISA), and for use in production of a rabbit polyclonal antibody. These reagents were utilized in development and optimization of an ELISA, which established the extent of exposure of free ranging raccoons to a newly described polyomavirus (RacPyV) [1]. Production of a polyclonal antibody has allowed for further characterization of RacPyV, including immunohistochemistry and immunocytochemistry techniques, in order to answer questions about pathogenesis of this virus.

**Specifications Table**

**Table:** 

Subject area	Biology
More specific subject area	Viral oncogenesis
Type of data	2 figures
How data was acquired	Protein Simple FluorChem E, BioTek ELx800
Data format	Raw
Experimental factors	NA
Experimental features	Recombinant protein production using baculovirus expression system and subsequent polyclonal antibody production and immunoblot analysis
Data source location	California and Georgia, United States
Data accessibility	Data is with this article

**Value of the data**

• Provides details about production of a recombinant viral protein from a non-human polyomavirus (raccoon polyomavirus, RacPyV).
• Recombinant protein provides for numerous downstream applications, including indirect ELISA and polyclonal antibody production.
• Subsequent polyclonal antibody derived from recombinant protein provides for a positive control on ELISA for this novel virus, for which negative and positive populations are not defined.

## 1 Data

Exposure to human polyomaviruses is widespread [2], but seroprevalence studies examining natural infection in wild animals are lacking, with the exception of a handful involving mouse polyomavirus [3]. Previous work has demonstrated that raccoon polyomavirus (RacPyV) is a novel polyomavirus involved in neuroglial tumor formation in raccoons [4]. However, basic characteristics of RacPyV, including distribution and seroprevalence have not been previously examined. In order to establish these characteristics, our group set out to develop an indirect enzyme linked immunosorbent assay (ELISA) [1].

## 2 Experimental design, materials and methods

### 2.1 Recombinant viral protein production

The entire RacPyV VP1 gene sequenced from tumor tissue (Rac 2) plus a terminal sequence encoding six histidines was cloned into the baculovirus expression vector pFastBac. Recombinant Baculovirus was generated using the Bac-to-Bac system (Life Technologies/Fisher Scientific, Illkirch, France). Tni (*Trichoplusia ni*) insect cells (Expression Systems, LLC, Davis, CA) were infected with recombinant Baculovirus at an MOI of 3. Insect cells were pelleted, lysed on ice in 1× cobalt buffer (0.3 M NaCl, 50 mM Na_2_HPO_4_ in milliQ water at pH 7.4) plus protease inhibitor (cOmplete^TM^ EDTA free protease inhibitor cocktail tablets, Roche). VP1 protein was purified by incubation overnight at 4 °C with HisPur cobalt resin (Thermo Scientific, Rockford, IL, USA) followed by washes with increasing concentrations of imidazole (10 mM, 20 mM, and 40 mM imidazole in PBS) and elution with 150 mM imidazole elution buffer. Protein elutions were then buffer exchanged overnight with PBS at 4 °C to remove imidazole. Purified virus like particles (VLPs) were then coated onto 96 well Maxi-sorp plates for ELISA and serosurvey of collected raccoon sera [1].

### 2.2 Electron microscopy

**Fig. 1:**
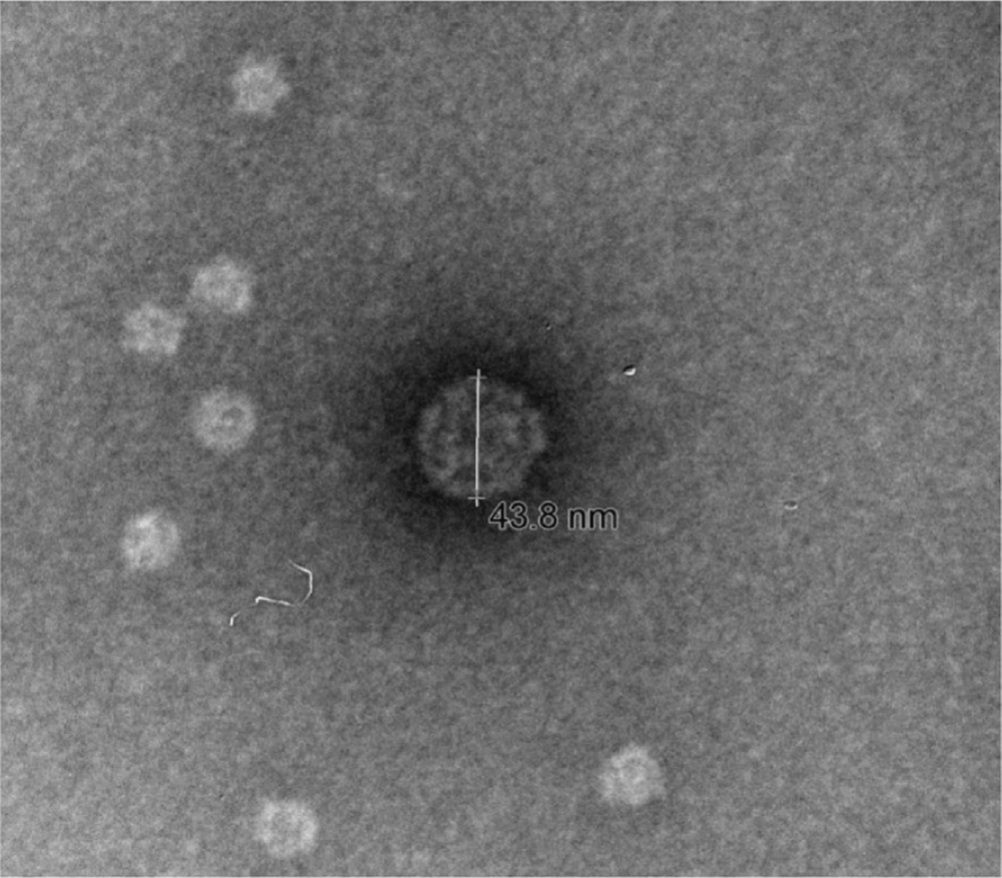
Virus-like particles from purified rRacPyV VP1 produced in Tni insect cells are the expected size for polyomaviruses (approximately 45 nm).

Presence of VLPs was confirmed by negative staining (direct) electron microscopy (Fig. 1). Briefly, purified PBS-exchanged protein elution was combined with 2% phosphotungstic acid (pH adjusted to 7.0 with NaOH) in a 1:10 ratio. A small drop of the solution was placed on a Formvar coated copper grid stabilized with evaporated carbon film, and the excess removed after 30 s. The prepared sample was observed at 80Kv on a Zeiss LEO900e transmission electron microscope at the California Animal Health and Food Safety Laboratory in Davis. Several particles ranging in size from 40 to 50 nm in diameter, consistent with the size of polyomavirus virions, were observed. Several smaller particles present in the photomicrograph are similar to particles previously reported in insect-cell based recombinant protein systems [5].

### 2.3 Polyclonal antibody production

Anti-VP1 polyclonal antibody was produced in a New Zealand white rabbit. Briefly, pre-immune serum was collected and 500 µg of purified recombinant VP1 protein was injected subcutaneously four times at two-week intervals. Serum was collected prior to each injection for a total of five samples (Pre-immune, bleed 1, bleed 2, bleed 3, bleed 4). Serum from bleed 2 was used as a positive control for the RacPyV ELISA [1].

### 2.4 Western blot analysis

**Fig. 2:**
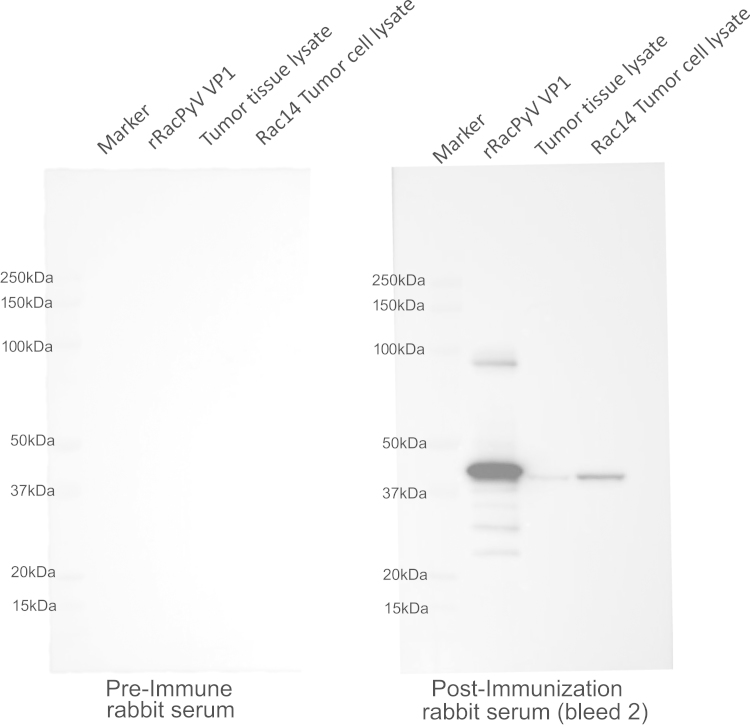
Western blot analysis of pre-immune serum collected from rabbit prior to injection with rRacPyV VP1 protein and of test bleed 2 serum (anti-VP1 polyclonal antibody. Immunoblot of the preimmune serum demonstrates no immunoreaction to any of the proteins loaded. Immunoblot of the polyclonal antibody demonstrates immunoreactivity to protein at 42 kDa, the predicted size of RacPyV VP1.

Western blot analysis was performed in order to verify binding specificity of our anti-VP1 polyclonal antibody. Protein was prepared for western blot analysis as follows: Purified PBS-exchanged protein elution containing recombinant (rRacPyV VP1) was quantified and loaded onto gels as described below. Tissue from neuroglial tumors was prepared by lysis using a dounce homogenizer followed by incubation of homogenate in RIPA lysis buffer with protease inhibitors (cOmplete^TM^ EDTA free protease inhibitor cocktail tablets, Roche). Pellets of the neuroglial tumor cell line developed from tumor tissue from Rac 14 (2014F12Rac1Mar) were thawed on ice, triturated, and lysed in RIPA lysis buffer with protease inhibitors. Samples were incubated on ice and intermittently vortexed for 20 minutes, and then were centrifuged at >10,000 rpm at 4° for 10 min. All protein samples were quantified using a modified Bradford assay (Biorad), with absorbance measured and concentration calculated against known concentrations of bovine serum albumin standards using a microplate reader (BioTek ELx800). Protein was loaded in the following amounts onto 4−12% NuPAGE^®^ Bis-Tris gels (Invitrogen, Carlsbad, CA) for electrophoresis: 0.25 μg of rRacPyV VP1 protein into lane 2; 60 μg of protein from lysate of tumor tissue (Rac 12 − 2013K15Rac1Yolo) was loaded into lane 3; 60 μg of protein from lysate of tumor cell line was loaded into lane 4 (Fig. 2). Electrophoresed protein was then transferred overnight at 4° at 15V to PDVF membranes, 0.45 μm pore size. Membranes were placed in 5% nonfat milk for 1h at room temperature to inhibit non-specific antibody binding. For confirmation of reactivity of the polyclonal antibody to RacPyV VP1 described above, membranes were then incubated with either pre-immune serum collected from the rabbit prior to injection with rRacPyV VP1, or with rabbit serum isolated following immunization against rRacPyV VP1. Both pre-immune and post-immunization sera were incubated at a dilution of 1:5000 in 5% milk at room temperature for 1 h. Primary antibodies from pre-immune and post-immunization sera were detected using goat anti-rabbit IgG HRP-conjugated antibodies at a concentration of 1:20,000 at room temperature for 1 h (Life Technologies). Following incubation with ECL substrate (Amersham), images of the immunoblots were obtained using a digital chemiluminescent detection system (Protein Simple). Accordingly, VP1 binding specificity of post-immunization rabbit serum was confirmed by the presence of a 42 kDa band (predicted size of RacPyV VP1) (Fig. 2).

**Fig. 3:**
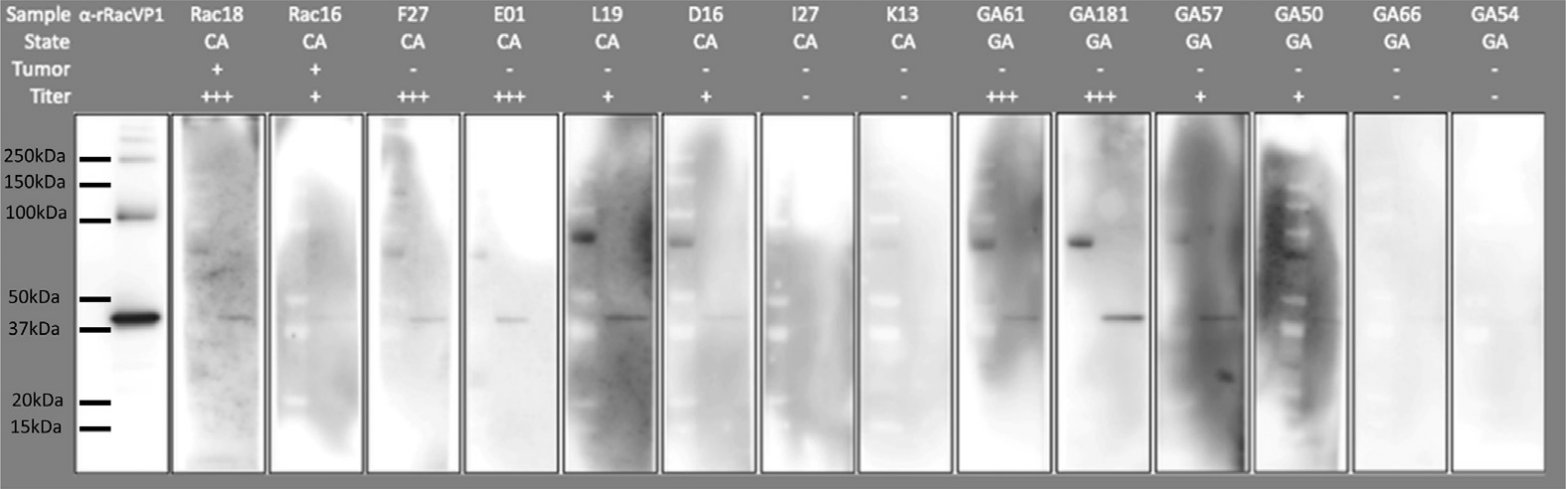
Western blot analysis of a subset of raccoon sera hybridized to rRacPyV VP1 protein. Tumor status and relative titer level are denoted in label. Rabbit anti-VP1 antibody serves as positive control (first column).

Western blot analysis was performed on a subset of raccoon sera alongside our polyclonal antibody to verify specificity of ELISA reaction (Fig. 3). Additionally, serum samples from raccoons in areas where neuroglial tumors have been diagnosed (California, CA) and in areas where no neuroglial tumors have been diagnosed in free-ranging raccoons (Georgia, GA) were analyzed. 0.25 μg of rRacPyV VP1 was loaded onto gels, electrophoresed, and transferred to membranes as described above. Three groups of sera based on ELISA results [1] were analyzed by western blot: (1) raccoons with high ELISA titers (≥51,200), (2) raccoons with low ELISA titers (≤3200), and (3) raccoons with negative ELISA results (Fig. 3). Membranes were incubated with sera from raccoons in CA and raccoons in GA at dilutions of 1:400 at room temperature for 1 h. Goat anti-raccoon IgG HRP-conjugated secondary antibody (Bethyl Laboratories) was incubated at room temperature for 1 h at a dilution of 1:5000. Sera that were positive by ELISA from raccoons with tumors (Rac18, Rac16) and without tumors (F27, E01, L19, D16, GA61, GA181, GA57, GA50) were immunoreactive to protein at 42 kDa, regardless of the geographic origin of the sample. None of the sera that were negative by ELISA were immunoreactive by western blot analysis. These analyses confirm specificity of immunoreactivity to protein of the predicted size for RacPyV VP1 in raccoon serum samples that were positive by ELISA and absence of immunoreactivity in samples that were negative by ELISA.
