# Supplementary material for: Production of a recombinant capsid protein VP1 from a newly described polyomavirus (RacPyV) for downstream use in virus characterization
Source: Data Brief. 2016 Feb 9;7:60–5. doi: 10.1016/j.dib.2016.01.070 (PMC4761659; doi:10.1016/j.dib.2016.01.070)
Supplement: Supplementary file 2 — Supplementary material [file mmc2.pdf]

Authorship & Conflicts of Interest Statement

Manuscript title: Production of a recombinant capsid protein VP1 from a newly described polyomavirus (RacPyV) for downstream use in virus characterization

AUTHORSHIP

All persons who meet authorship criteria are listed as authors, and all authors certify that they have participated sufficiently in the work to take public responsibility for the content, including participation in the concept, design, analysis, writing, or revision of the manuscript. Furthermore, each author certifies that this material or similar material has not been and will not be submitted to or published in any other publication.

In the table below, indicate the specific contributions made by each author (list the authors' initials followed by their surnames, e.g., Y.L. Chang) to the submitted manuscript. A check mark (√) must appear against the name of each author at least once in each of the three categories below.

Author name	Category 1			Category 2	Category 3
	Conception and design of study	*Acquisition of data (laboratory or clinical)*	*Data analysis and/or interpretation*	*Drafting of manuscript and/or critical revision*	*Approval of final version of manuscript*
EXAMPLE: Y.L. Chang	√		√	√	√
ME Church	X	X	X	X	X
FN Dela Cruz		X	X		X
K Kim		X	X		X
M Persiani		X	X		X
LW Woods		X	X		X
PA Pesavento	X	X	X	X	X
KD Woolard	X	X	X	X	X

Acknowledgments

All persons who have made substantial contributions to the work reported in the manuscript (e.g., technical help, writing and editing assistance, general support), but who do not meet the criteria for authorship, are named in the Acknowledgments and have given us their written permission to be named. If we have not included an Acknowledgments in our manuscript, then that indicates that we have not received substantial contributions from non-authors.

CONFLICTS OF INTEREST

A conflict of interest occurs when an individual's objectivity is potentially compromised by a desire for financial gain, prominence, professional advancement or a successful outcome. *ASJSUR* Editors strive to ensure that what is published in the Journal is as balanced, objective and evidence-based as possible. Since it can be difficult to distinguish between an actual conflict of interest and a perceived conflict of interest, the Journal requires authors to disclose all and any potential conflicts of interest.

Section I

The authors whose names are listed immediately below certify that they have NO affiliations with or involvement in any organization or entity with any financial interest (such as honoraria; educational grants; participation in speakers' bureaus; membership, employment, consultancies, stock ownership, or other equity interest; and expert testimony or patent-licensing arrangements), or non-financial interest (such as personal or professional relationships, affiliations, knowledge or beliefs) in the subject matter or materials discussed in this manuscript.

Author names: Molly E. Church, Florante N. DelaCruz Jr., Kevin Kim, Michele Persiani, Leslie W. Woods
Patricia A. Pesavento, Kevin D. Woolard

Section II

The authors whose names are listed immediately below report the following details of affiliation or involvement in an organization or entity with a financial or non-financial interest in the subject matter or materials discussed in this manuscript. Please specify the nature of the conflict on a separate sheet of paper if the space below is inadequate.

Author names:	Details of the conflict(s) of interest:
