# Supplementary material for: Production of a recombinant capsid protein VP1 from a newly described polyomavirus (RacPyV) for downstream use in virus characterization
Source: Data Brief. 2016 Feb 9;7:60–5. doi: 10.1016/j.dib.2016.01.070 (PMC4761659; doi:10.1016/j.dib.2016.01.070)
Supplement: Supplementary file 1 — Supplementary material [file mmc1.docx]

Dear Dr. Wang,

Please find enclosed a manuscript entitled “Production of a recombinant capsid protein VP1 from a newly described polyomavirus (RacPyV) for downstream use in virus characterization” for consideration in the journal Data in Brief.

The work described in this manuscript, namely the production of a recombinant viral capsid protein to raccoon polyomavirus (RacPyV), and the characterization of its downstream use in an ELISA, represents a significant expansion in the reagents available for the characterization of this newly characterized polyomavirus. While characterization of this virus is still ongoing, it has generated considerable interest, resulting in several recent publications, including the Journal of Virology (PMID 25165109) and as a complimentary manuscript in the Journal Virology (Church et. al. *In Press*).

We feel that the methodology of this investigation is of interest not only to basic researchers, but also to wildlife biologists and veterinary medicine. As this manuscript details the initial production of recombinant protein in an emerging disease alongside its incorporation into serological surveillance and *in vitro* characterization, it provides an in depth summary of an investigation into a novel virus.

All authors testify to the validity of the results, and to its originality. None of this work has been previously published. Thank you most genuinely for your consideration of our manuscript for publication in your journal.

Kind Regards,

Kevin D. Woolard

Assistant Professor, UC Davis School of Veterinary Medicine

Attachments:

*Manuscript*

*Figures*

*Response to reviewers*
